# Decompression Surgery Options for Metastatic Cervical Spine Lesions

**DOI:** 10.7150/jca.81594

**Published:** 2023-04-01

**Authors:** Liubov Gorbacheva, Mikhail Potapov, Vadim Taran

**Affiliations:** 1 Kuban State Medical University, Krasnodar, Russian Federation; 2 Kemerovo State Medical University, Kemerovo, Russian Federation

**Keywords:** metastatic lesion of the spine, decompression surgery, vertebral stabilization, neurological disorders, regression of pain syndrome.

## Abstract

**Background**: Metastatic spinal lesions occur in 70% of patients with incurable cancer, and the most common site for bone metastases is the spine. Over the last decade, medical science has made significant progress in treating tumor damage to the spine. The study examined the efficacy of decompression surgery for patients with metastatic cervical spine lesions contributing to spinal cord compression.

**Methods**: The study enrolled 38 patients (27 females and 11 males, average age of 61.35±8.49 years) with metastatic cervical spine lesions resulting in cord compression relieved with surgery. Patients experienced improvement in pain and motor within one month of surgery addressing cervical metastatic disease.

**Results**: Complete or partial regression of pain syndrome 10 days after surgery was observed in 26 (68.4%) patients, one month later - in 33 (86.8%) patients, one year later - in 35 (92.1%) patients. Regression of neurological symptoms on the 10th day after surgery was observed in 8 (21.1%) patients, one month later - in 21 (55.3%) patients, one year later - in 34 (89.5%) patients. Two patients died between 3 and 12 months after surgery, having a worsening of their neurological status and pain syndrome.

**Conclusions**: Decompression surgeries for metastatic lesions of the cervical spine with spinal cord compression resulted in effective reduction of pain and neurological dysfunction.

## Introduction

Diagnosing and treating metastatic lesions of the skeletal system is one of the most challenging and problematic sections of modern clinical oncology 1. The most common site for bone metastases is the spinal cord. Bone metastases are a frequent manifestation of generalized cancer. Spinal metastases occur in an average of 10% of patients with a cancer diagnosis: the thoracic (70%) and lumbar (20%) regions are most frequently affected. The pathological process is localized less frequently (10%) in the cervical spine. At the same time, up to 70% of patients survive in a five-year period; therefore, bone cancer is not a type of cancer with high mortality rates 2-4.

New chemotherapy drugs have been introduced (Alkylating agents, Antimetabolites, Plant alkaloids, Antitumor antibiotic), and radiotherapy techniques and surgical treatment methods have improved considerably 5, 6. The primary goals that physicians must strive to achieve in treating spinal tumors and metastases are the relief of pain syndrome and the restoration of neurological stability in the affected segment 7, 8. Surgery in patients with metastatic spinal lesions is required if they suffer from nerve root pain syndrome with spinal instability, intractable pain, increasing spinal cord compression, and pathological vertebral fracture with spinal cord compression 6, 9.

Decompression surgery is a procedure performed to relieve pressure on nerve roots or the spinal cord. In the cervical spine, an anterolateral retropharyngeal access is used to provide anatomically the most accessible and physiologically sound access to the spinal column with minimal crossing of the supporting muscles of the neck. Anterior cervical somatectomy (in some cases multistage) is performed in several variants depending on the biological characteristics and size of the tumor: a) anteromedial or partial somatectomy; b) medial somatectomy including resection of the middle part of the vertebral body in cases of complete posterior anterior lesion; c) total removal of the vertebral body (total somatectomy).

In addition to anterior somatectomy, extended somatectomy (removal of the entire anterior segment) and total vertebrectomy (removal of the anterior segment, arch, and articular tuberosities) are performed when necessary. In some cases, decompression can be achieved by simple extirpation (excochleation) of the metastatic mass without resection of the adjacent vertebral body 10.

Approaches to surgical treatment of spinal metastases of different multiple lesions. Multiple metastases surgery is aimed at stabilizing the spinal column and decompressing the nerve structures from tumor masses, in accordance with the oncological principles of ablation. Long-term results after such surgical interventions, taking into account the impact of systemic therapy, are of clinical interest. Surgical treatment of metastatic lesions of the vertebra, particularly of the cervical spine, is a complex and pressing problem of neuroorthopedics 11.

Multilevel fixation of the spine using minimally invasive procedures is indicated for patients diagnosed with diffuse spinal metastasis, as it was shown in a sample of 24 patients. In this case, minimization of blood loss occurs due to intervention through the skin 12. In the postoperative period, stereotactic radiotherapy (SBRT) is an effective therapy for the treatment of spinal metastases 13. Anterior transcorporeal access was shown to be effective for resection of a cancerous tumor in the ventral cervical region and decompression of the cervical spinal cord. The minimally invasive is the peculiarity of this technique. Decompression surgery showed reduced mortality due to the low probability of complications after surgery. The factors that reduce short-term mortality also include chemotherapy 14.

So far, conservative treatment is the main method of treatment of spinal metastases. That is why there is a need for research, which could expand the knowledge about the possibilities of applying various methods of surgical treatment for metastatic lesions of the spine. Of separate interest are the studies of the neurological status as well as the neurological changes occurring in patients in the postoperative period. The aim of the study was to examine the effectiveness of decompression surgeries in patients with metastatic lesions of the cervical spine with spinal cord compression. It was found that decompression operations in metastatic lesions of the cervical spine with spinal cord compression might lead to an effective reduction of pain syndrome and neurological dysfunction. At the same time, decompression in cervical metastases might lead to a decrease in pain syndrome and neurological dysfunction. Consequently, this study compared the effectiveness of operational decompression methods of anterior and posterior access.

## Material and methods

Study design, protocol, and the form of informed voluntary consent for participation in the study were reviewed and approved by the Commission on Bioethics of Kuban State Medical University (protocol No 2035180, 02/02/2014) and has been carried out in accordance with Declaration of Helsinki. All participants gave their written informed consent for participate.

A total of 38 patients with metastatic lesions of the cervical spine with spinal cord compression were included in the study (2014-2020), of whom 27 (71.1%) were women and 11 (28.9%) were men. Patients' age ranged from 46 to 78 years (average age was 61.35±8.49 years). All patients received treatment in the same clinic.

All patients underwent decompression surgery. The small sample size is due to the fact that not all patients agreed to sign the collaboration agreement and not all of the patients who agreed met the study inclusion criteria. Fifteen patients (9 women and 6 men) refused to participate in the study. The age of the patients was due to the fact that cancer is more common in the elderly than in the young. Since the study was based on random assessment, the sample randomly included a larger number of women being treated at the oncological dispensary. The bone cancer mostly occurs among young people (men) of 20-30 years of age. The elderly people included in the sample were being treated in an oncological dispensary at the time of the study. Localization of foci: lumbar spine in 14 patients, thoracic spine in 24 patients.

Patients were selected according to inclusion and exclusion criteria (Table 1).

The level of neurological disorders was assessed in accordance with the Frankel H. L. classification. The distribution of patients according to the severity of neurological disorders is shown in Table 2. The uneven distribution of patients was due to their division into groups according to the Frankel classification.

**Figure 1:**
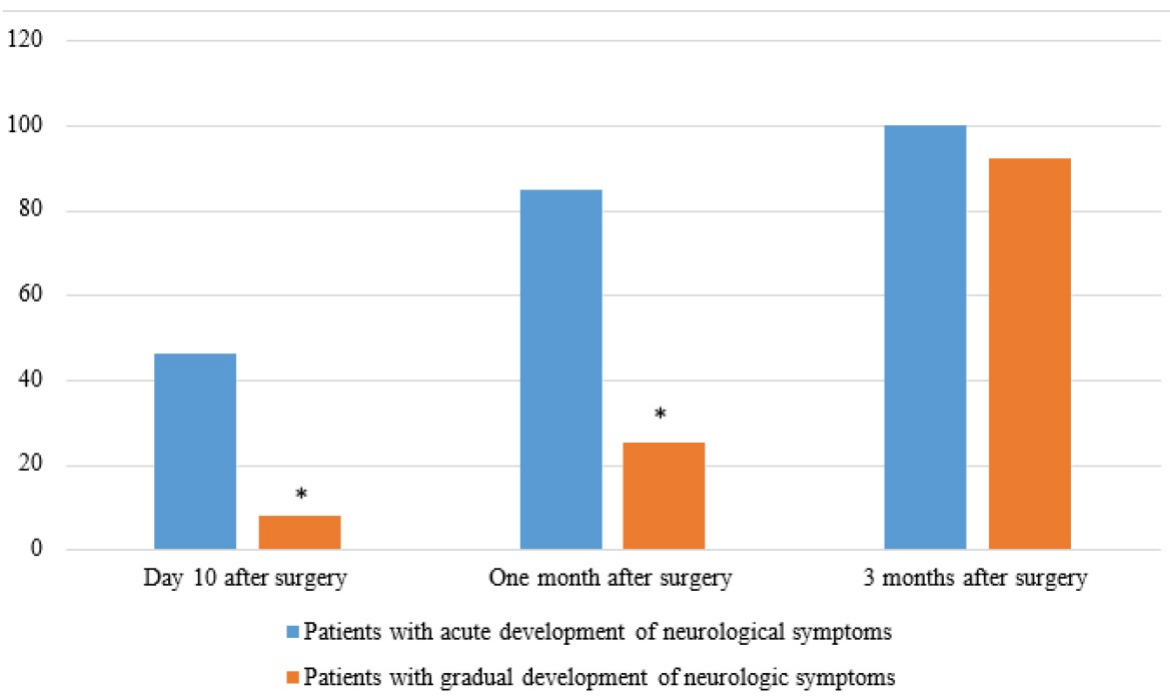
Proportion of patients undergoing neurological regression after surgery due to changes in neurological symptoms Note* - the difference is statistically significant between patients exhibiting acute and progressive neurological symptoms (p<0.05)

The most frequent localization of the primary lesion in the studied patients were: breast cancer and gastrointestinal cancer (GIT) in women, kidney, lung and GIT cancer - in men (Table 3, Figure 1).

### Surgery

Surgery consisted of operative decompression performed: anteriorly in 27 (71.1%) patients, and posteriorly in 11 (28.9%) patients (see Table 2).

In all cases the foci of the primary tumor were removed, except for 4 cases of posterior access, when decompression of the spinal cord by laminectomy was applied. Patients did not receive radiotherapy/chemotherapy after surgical treatment.

### Follow-up

Before surgical intervention and 10 days, 1, 3, 6, 12 months after surgery patients underwent:

- detailed physical examination (1 stage);

- evaluation of neurological status (1 stage);

- laboratory examination of blood parameters (general clinical blood test, determination of blood glucose, urea, creatinine, total protein, total bilirubin, activity of liver transaminases, lipidogram and coagulogram). The next stage was surgery, and finally, the third stage was the postoperative examination of patients.

This study was an evaluation of the subaxial spine. The access is chosen based on radiological data and the predominant area of tumor growth in the vertebra, as well as depending on the general condition of the patient (anesthetic risk determines the invasiveness of the intervention). Laboratory tests were performed as mandatory methods of preoperative examination of patients. The main goal of surgical intervention in the studied category of patients is to restore the support ability of the cervical spine, regression of neurological disorders and pain syndrome, and the intervention itself is compulsory. Surgical methods were designed to relieve, among other things, the pain syndrome; therefore, no systematic therapy was performed. Anesthesia risk levels were determined according to the ASA classification. The prescription of glucocorticosteroids (GCS) was reasonable for patients with signs of spinal cord compression.

Magnetic resonance imaging of the spine and scanning with radioactive technetium were performed before surgery and 12 months after surgery. Before surgery, 3, 6 months, and a year after surgery, patients underwent radiography of the spine. The limitation of this study was a small sample of patients, their gender (the majority were women), and age (elderly people). Therefore, further research is required.

### Statistical analysis

The processing of statistical data was completed using the SPSS 13 program. The following methods of variation statistics have been used: Student's t-test, Fisher's F-test, and Mann-Whitney U-test. The Fisher's 2 x 2 test was applied to compare quality features. The difference was regarded as statistically significant at p < 0.05.

## Results

### Postoperative pain relief

Already in the first days of the postoperative period, the analgesic effect of surgical intervention was observed in the studied patients, which was manifested by the regression of pain syndrome in a significant part of the patients (Table 4). The severity of pain syndrome was assessed based on patients' readings.

### Postoperative/recovery of neurological function

Positive dynamics of surgical treatment were also observed in the regression of neurological disorders (Table 4). This is reflected in a consistent decrease in the number of patients with neurological disorders and a decrease in the number of patients with a severe pain syndrome. It is worth noting that the regression of neurological disorders occurred with greater intensity and earlier in patients who had acute development of motor disorders in the preoperative period. Neurological condition improved by an average of 2 points. Of the Frankel A group, only one patient showed improvement.

### Intraoperative and postoperative complications

Unfortunately, a legitimate companion of invasive oncologic surgery is acute massive surgical blood loss, which is still a major problem for both surgeons and critical care medicine specialists. Bleeding can be so severe, exceeding by several times the estimated circulating blood volume that it poses a serious threat to a patient's life and sometimes, despite the extreme effort and resources of the operating room and intensive care unit personnel, the patient cannot be saved.

The following intraoperative/perioperative complications occurred during anterior cervical surgery: massive blood loss in 2 (7.4%) patients and dural sac lesions with liquorrhea in 3 (11.1%) patients; 10 (37.0%) patients had difficulty swallowing.

Postoperative complication included: neurological deterioration in 3 (11.1%) patients operated anteriorly and 1 (9.9%) patient operated posteriorly (p > 0.05). Spinal instability was noted in 3 (27.3%) out of 11 patients having posterior operations. The overall complication rate was 18.4%. Thus, patients, especially those who had had the back operations, most frequently experienced the spine problems. Apparently, regardless of the surgery type (anterior or posterior), some patients had deterioration in their neurological status, although in most cases both the status and the pain syndrome showed improvement. This indicates the effectiveness of the applied operating techniques.

Two patients died between 3 and 12 months after surgery. The cause of death in all cases was cancer (local tumor recurrence).

## Discussion

The study focused on the efficacy of surgical therapy in 38 patients with metastatic lesions of the cervical spine with spinal cord compression. The primary purpose of the surgical procedure in the patients studied was to restore the support capacity of the cervical spine and diminish neurological disorders, including pain syndrome 15-17. The surgical techniques (anterior and posterior access surgeries) used in patients with metastatic lesions of the cervical spine with spinal cord compression were effective with respect to the regression of pain syndrome and neurological disorders, as evidenced by their regression dynamics in the first month after surgery 18. Anterior access surgeries were associated with a higher incidence of intraoperative (liquorrhea, massive blood loss) and early postoperative complications compared to posterior access, but they did not affect the outcome of the surgical intervention in any way 19.

Several reports have been published on the effect of surgical treatment on life expectancy for spinal metastases. Jackson et al. 20 reported a mean survival of 14.1 months after various decompressive interventions. Prabhu et al. 21 showed that 37% of patients had an average postoperative survival of 11.5 months (8.7-21.4 months). Quraishi et al. 22 had a median survival of 12.3 months. Progress in the surgical technique of spinal reconstruction led to a paradigm shift toward conditionally radical operations with complete removal of the neoplasm. Kato et al. 23 analyzed the results of block resections of spinal metastases, where the median survival rate was 130 months.

In the late postoperative period, orthopedic complications - spinal instability - were observed in 3 patients (out of 11) who underwent posterior access surgery. These patients underwent secondary surgical intervention. Lumbar and cervical spine instability was treated with spondylodesis. Different variants of the operation are used for this procedure. This can be either open surgery or minimally invasive techniques. The task of surgical treatment is to stabilize the vertebrae of the affected spine, and this is done by means of special devices. The fixation elements hold each vertebra in a physiological position, aligning the entire spine. The duration of surgery and length of hospital stay depend on the volume of surgery and are determined individually depending on the clinical situation. This paper presents data directly related to the localization and surgical intervention to tumors in the spinal column. The authors took into account the patient's condition during examination and tests, including the burden on other organ systems. Without this, it would have been impossible to perform surgery, for example, taking into account the anesthesia risk.

Tokuhashi scale was used. Tokuhashi scale seems to be the most comprehensive due to the fact that it includes a block assessing the severity of neurological manifestations of spinal cord compression, allowing a strategic decision to treat the patient directly at the initial consultation. For the two deceased patients, the scale was 8 points (1 patient) and 11 points (1 patient). The remaining patients scored 13 or higher. The authors did not exclude deceased patients because their deaths occurred rather late in the postoperative period.

The present study showed a high survival rate of patients after the performed surgery, 2 people died within 3-12 months after surgical intervention. According to Atanasiu et al. 24, in lower cervical lesions, anterior access is recommended even for C3 (glossopharyngeal access) or cervico-thoracic lesions. In this case, patient management ranges from palliative nonoperative to surgical treatment 25. In the most severe cases, in patients with C1, C2, posterior access may be recommended 26. Neurological complications and increasing pain syndrome may be grounds for immediate surgical intervention 27. The benefits of posterior access for cervical metastases can alleviate pain, stabilize the spine, and improve the patient's quality of life 28. Life expectancy and quality of life can be improved by concomitant medication 29-31.

On average, overall survival in patients with small-cell lung cancer metastases varies between 6 months for 60% of patients, while for patients with kidney or prostate cancer metastases the overall survival is 1.5-2 years. Provided a good response to targeted therapy, patients with kidney cancer metastases live more than 5 years. In the present case, the authors attributed the low mortality rate to timely surgical intervention.

### Studies on efficacy of surgical treatment of cervical metastatic disease

Multiple studies document the effectiveness of surgery for metastatic cervical spine lesions (Table 5).

In the Vazifehdan et al. study 9, as in the present study, the primary tumor focus in men was lung and kidney cancer, in women - breast cancer. Complete or partial regression of pain syndrome occurred in 21 patients after surgical intervention; complete (in 7 patients) or partial (in 2 patients) regression of neurological disorders occurred in 9 patients; the complication rate was 20.8%. The results of Yang et al. 10 and Lei et al. 15 studies also demonstrate the effectiveness of decompression surgeries for cervical spine metastatic lesions; these studies are generally consistent with the present study findings. The present study not only confirmed the effectiveness of decompression surgery for metastatic lesions of the cervical spine, but also showed that with different access techniques (anterior and posterior) did not cause statistical differences in factors such as neurological status and pain syndrome. Most patients had these parameters improved, while they worsened for a minority. Thus, decompression surgery leads to an improvement in neurological status and a reduction of pain syndrome.

## Conclusions

Decompression surgeries for metastatic lesions of the cervical spine with spinal cord compression resulted in effective reduction of pain and neurological dysfunction. Thus, decompression surgeries for metastatic lesions of the cervical spine with spinal cord compression are effective and lead to complete or partial regression of neurological disorders. One month after surgery, partial regression of neurological symptoms was achieved in 12 (31.6%) patients, complete regression was achieved in 9 (23.7%) patients, 3 months later - in 26 (68.4%) and 10 (26.3%) patients, respectively. Also, complete regression of pain syndrome was achieved 10 days after surgery in 20 (52.6%) patients, partial - in 6 (15.8%) patients, a month later pain syndrome partially persisted in 3 (7.9%) patients, a year later - in 1 (2.6%) patient.

Corporodesis with a carbon implant is required during spinal cord decompression, which allows achieving a reliable bone-carbon block as early as 3 months after surgical intervention without neuroorthopedic abnormalities. The surgical techniques used are not associated with the occurrence of spinal and hypodynamic complications, since early patient rehabilitation is allowed. The perspective of the decompression surgery method is determined by low indicators of pain syndrome and improvement in neurological status. In the future, an additional research on the issue is required to transcend one of the study limitations related to the small sample size. Further research may also be connected with the identification of possible gender differences when comparing the effectiveness of anterior and posterior access, regarding patients' age.

## Funding

This research did not receive any specific grant from funding agencies in the public, commercial, or not-for-profit sectors.

## Ethics Committee Approval and Patient Consent

Study design, protocol, and the form of informed voluntary consent for participation in the study were reviewed and approved by the Commission on Bioethics of Kuban State Medical University (protocol No 2035180, 02/02/2014) and has been carried out in accordance with Declaration of Helsinki. All participants gave their written informed consent for participate.

## Figures and Tables

**Table 1:** List of inclusion and exclusion criteria

Inclusion criteria	Exclusion criteria
metastatic lesion of the cervical spine with spinal cord compression; the need for surgical intervention; age over 18 years; signed informed consent of the patient to participate in the study	age under 18 years; primary tumor of the cervical spine; localization of metastatic lesion in the thoracic or lumbar spine; acute myocardial infarction; acute cerebral circulation disorder; mental disorder; lack of consent.

**Table 2:** Distribution of investigated patients according to the severity of neurological disorders (according to Frankel H. L.), the development of local pain syndrome and neurological disorders, and the nature of surgical interventions

Characteristics	Absolute number	%
Distribution of the studied patients according to Frankel's classification		
Group A	3	7.9
Group B	8	21.0
Group C	21	55.3
Group D	6	15.8
Group E	0	0.0
Characteristics of the development of local pain syndrome and neurological disorders		
Acute development	13	34.2
Gradual development (over 2-3 weeks)	25	65.8
Distribution of patients according to the nature of surgical intervention		
Posterior decompression surgeries	11	28.9
Anterior decompression surgeries, including:	27	71.1
Stabilization of 2 vertebral segments	19	50.0
Stabilization of 3 vertebral segments	15	39.4
Stabilization of more than 3 vertebral segments	4	10.6

**Table 3:** Distribution of patients by primary focal spot in metastatic cervical column tumors

Localization of the primary focus	Total (n=38)		Females (n=27)		Males (n=11)		Р
	Abs.	%	Abs.	%	Abs.	%	
Breast cancer	21	55.3	21	77.8	-	-	<0.01*
Kidney cancer	6	15.8	2	7.4	4	36.4	0.04*
Lung cancer	3	7.9	-	-	3	27.3	0.02*
Gastrointestinal cancer	7	18.4	4	14.8	3	27.3	0.23
Bladder cancer	1	2.6	-	-	1	9.0	0.29

Note. * the difference is statistically significant between female and male patients (p<0.05).

**Table 4:** Dynamics of regression of pain syndrome and neurological disorders in patients with cervical spine metastases after surgical intervention

Characteristics, number of people	10 days after surgery	One month after surgery	One year after surgery
Number of people with regression of pain syndrome, absolute number (%)	26 (68.4 %)	33 (86.8 %)*	36 (92.1 %)*
Number of people with regression of neurological disorders, absolute number (%)	8 (21.1 %)	21 (55.3 %)*	34 (89.5 %)*/**

Note. * - differences are statistically significant compared with the indicator on 10th day after

**Table 5:** Results of other research on the effectiveness of decompression surgeries for metastatic lesions of the cervical spine

Author, country	Year	Participants	Pathology under study	Type of intervention	Median survival rate	Frequency of complications
Vazifehdan et al. 9 Germany	2017	24 patients: men - 14, women - 10; age 54-89	metastatic lesion of the cervical spine	Decompression spine surgeries: posterior - in 15 patients, anterior - in 9 patients.	14.8 months	20.8%
Yang et al. 10 China	2017	39 patients: men - 29, women - 10; age 18-80	atlantoaxial metastases	Tumor decompression and resection by posterior access - in 38 patients, anterior - in 1 patient	18 months	12.8%
Lei et al. 15 China	2015	95 patients: men - 55, women - 40; age 29-69	metastatic spine lesions, including cervical spine lesions in 19 patients	Posterior decompression surgeries	11.5 months	18.9%; metastatic lesions of the cervical vertebrae - 26.3%
